# Incidence of Encountering the Infrapatellar Nerve Branch of the Saphenous Nerve During a Midline Approach for Total Knee Arthroplasty

**DOI:** 10.5435/JAAOSGlobal-D-19-00160

**Published:** 2019-12-12

**Authors:** Nicholas F. James, Arun R. Kumar, Benjamin K. Wilke, Glenn G. Shi

**Affiliations:** From the Department of Orthopedic Surgery, Mayo Clinic, Jacksonville, FL.

## Abstract

The infrapatellar nerve branch (IPNB) of the saphenous nerve supplies cutaneous sensation to the anterolateral knee. Given its location and variable course, the IPNB is suspected to be at risk of injury with commonly used incisions around the knee. Nerve injury may lead to painful neuroma formation. To our knowledge, no study has evaluated the incidence at which the IPNB is encountered during the anterior approach incision for a routine total knee arthroplasty (TKA). The purpose of this study was to see whether the general joint arthroplasty surgeon can identify and examine the location of the IPNB encountered during primary TKA and to determine whether these branches would be transected during a standard medial arthrotomy.

**Methods:**

Seventy-three patients (76 knees) underwent primary TKA using a standard midline approach with a medial parapatellar arthrotomy. The IPNB was identified, and the distance was measured from the inferior pole of the patella to the point where the nerve crossed the medial border of the patellar tendon. This distance was then compared with the length of the arthrotomy in the same knee to determine whether the nerve would be transected.

**Results:**

The IPNB was encountered in all knees with a mean distance of 2.82 cm (95% confidence interval, 2.58–3.06) distal to the inferior pole of the patella during the arthrotomy. Patient characteristics including sex, height, and body mass index were not markedly associated with nerve location.

**Conclusion:**

The IPNB of the saphenous nerve is at risk for injury and routinely encountered by the general orthopaedic surgeon during a standard TKA medial parapatellar approach without the aid of magnification or dye.

Total knee arthroplasty (TKA) is a widely accepted orthopaedic treatment for painful end-stage arthritis with often reproducible improvement in pain, function, and quality of life metrics.^1^ The number of procedures being performed is projected to increase with the increase in the aging population.^2^ Most patients are satisfied with the outcome of the procedure; however, nearly 20% are dissatisfied with their results^3^ because, in large part, of continued pain from intra- or extra-articular causes.^4^ A lesser known, but previously described, extra-articular source of pain is neuroma formation after injury to the infrapatellar nerve branch (IPNB) during a standard approach for TKA.^5^

The IPNB, which arises from the saphenous nerve as it exits the adductor canal, provides cutaneous sensation to the anterior knee.^6,7^ Variations of its course and branching pattern have been described.^8^ Most reported one to three sub-branches of the IPNB that cross the knee and terminate to supply cutaneous sensation to the anterolateral knee.^9,10,11,12^

Iatrogenic injury to the IPNB has been suspected within the orthopaedic literature across several types of knee surgical procedures, including TKA.^8,13-15^ Injury to this nerve can cause clinical symptoms ranging from mild to moderate hypesthesia to severe pain, which can lead to postoperative stiffness and poor knee function.^16,17^

Although previous research has been performed using cadaveric dissection or ultrasonography evaluation of the course of the nerve,^9,10,11,12,18,19^ to our knowledge, no study has evaluated the incidence with which the nerve is encountered and transected during a standard approach for TKA.

The primary purpose of this study was to determine the incidence of encountering the IPNB of the saphenous nerve during the anterior knee approach for a primary TKA with a medial parapatellar arthrotomy. A secondary purpose was to identify any patient characteristics associated with the IPNB location.

## Methods

After institutional review board approval, we performed a prospective study to evaluate the incidence of encountering the IPNB during primary TKA. Patients undergoing a revision procedure or those who had previous midline incisions were excluded from the study. A single surgeon (B.K.W.) performed all procedures to maintain consistency.

**Figure 1:**
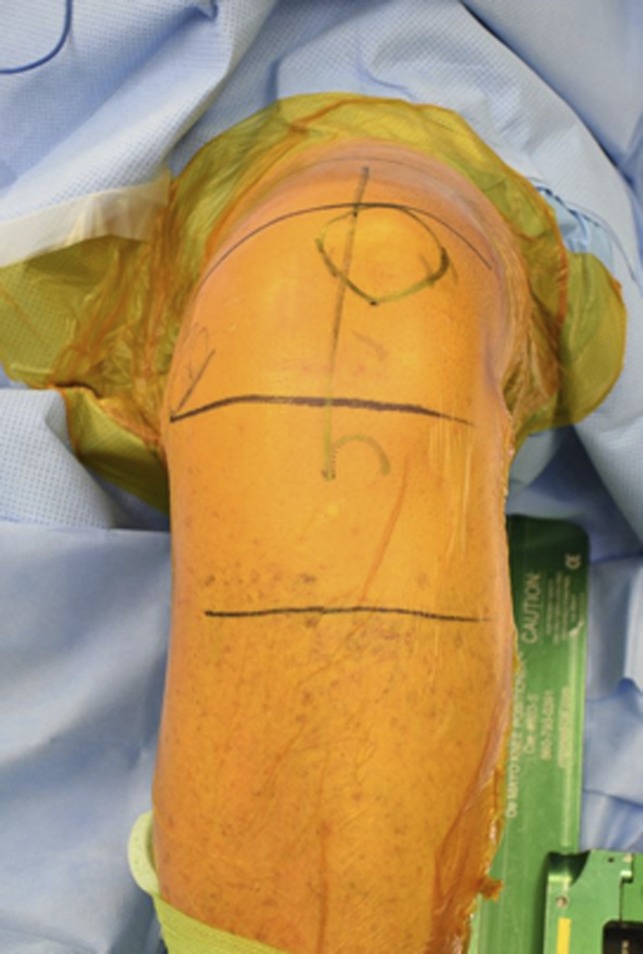
Photograph showing skin markings for the planned incision for total knee arthroplasty.

**Figure 2:**
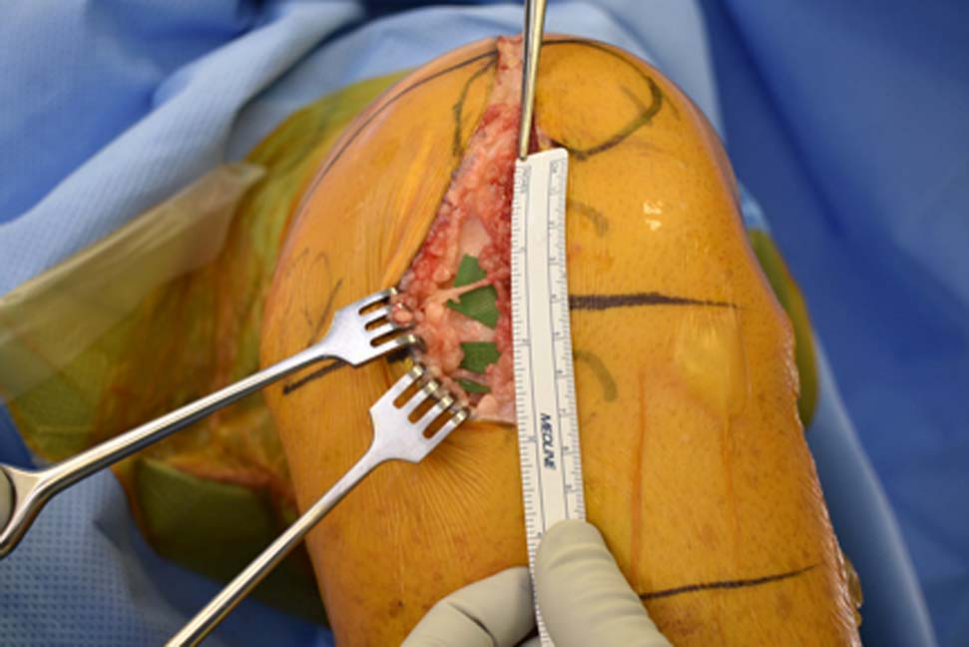
Photograph showing multiple branches of the infrapatellar nerve measured from the inferior aspect of the patella.

A standard midline approach with a medial parapatellar arthrotomy was used for all procedures (Figure 1). During the exposure, the inferior half of the incision was carefully dissected to determine whether the IPNB was present in the wound. The IPNB was identified by visual inspection as a nerve located in the subcutaneous tissue that ran perpendicular to the incision (Figure 2). If multiple branches were found, the largest branch was chosen as the primary nerve branch and measurements were made from this branch. All measurements were performed with a readily available sterile steel surgical ruler. Neural tissue was confirmed in all cases by the existence of fascicles.

**Figure 3:**
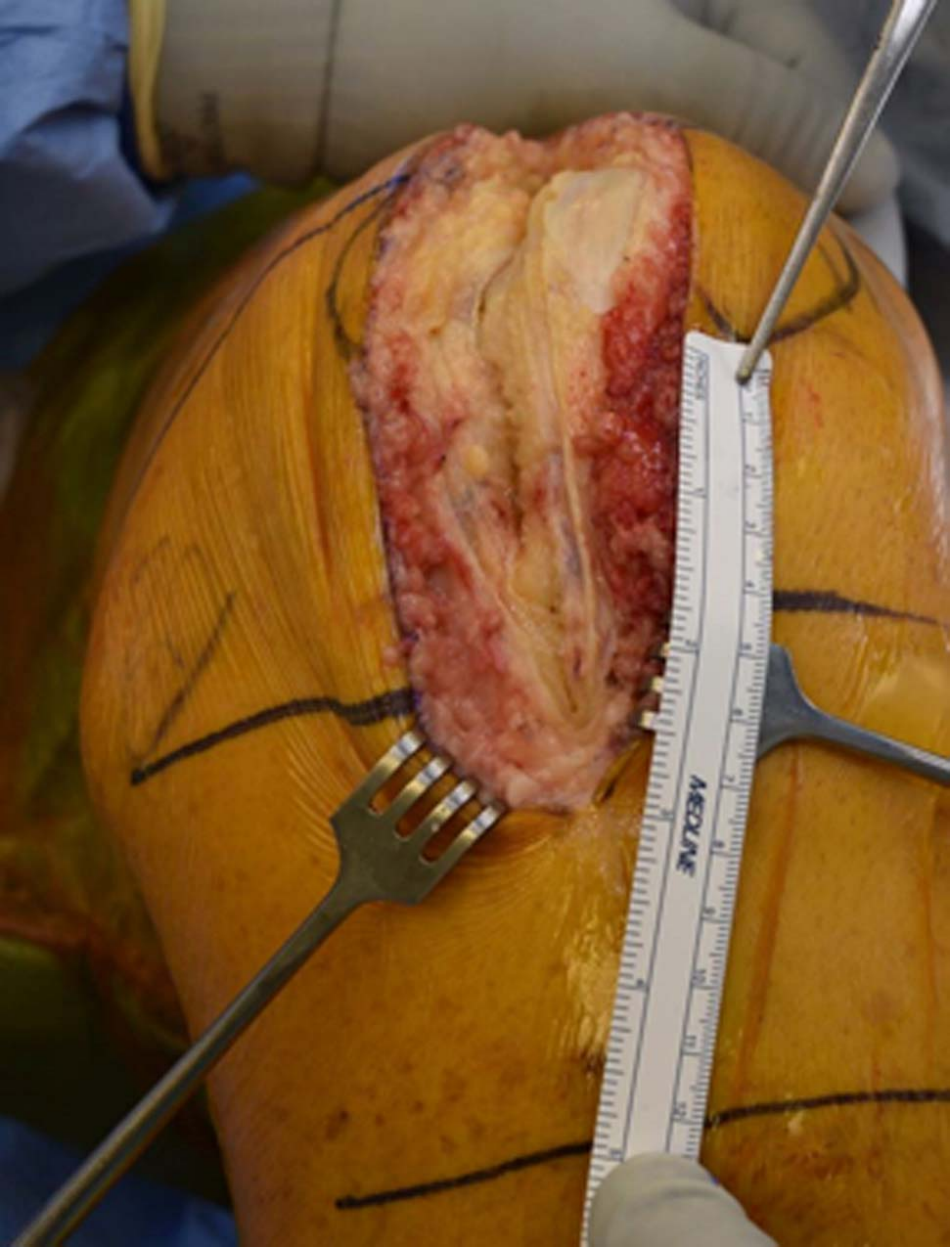
Photograph showing the distal extent of the arthrotomy in relation to the inferior pole of the patella.

Arthrotomy was then carried out sharply using a scalpel along the medial parapatellar border down to the level of the tibial tubercle. The distance that the arthrotomy extended from the inferior pole of the patella was then recorded (Figure 3), and the mean distance for all patients was calculated. Baseline demographics collected included age, sex, side of surgery, height, and body mass index (BMI).

A 2-tailed *t*-test was used to compare dependent continuous variables with independent categorical variables. Statistical significance was identified with a *P* value less than 0.05. The Pearson correlation coefficient was calculated to identify a correlation between continuous variables. Linear regressions were used to identify demographic characteristics as predictors of the location of the IPNB and the extent of arthrotomy.

## Results

Patient demographics are listed in Table 1. Seventy-three patients (76 knees) were included in our analysis. Forty-four (60.3) of the patients were women, with a mean (SD) age of 68.8 (9.7) years. Forty (52.6) of the knees were right knees. The mean (SD) height of the patients was 168.7 (10.5) cm, and mean (SD) BMI was 31.9 (5.1).

**Table 1:** Demographic Data

Total no. of patients	73
Males	29
Females	44
Average age (SD)	68.8 (9.7)
Range	48-85
BMI (SD)	31.9 (5.1)
Range	20.7-47.4
Height (SD)	168.7 (10.5)
Range	149-190
Total no. of knees	76
Right	40
Left	36
Nerve encountered in approach (%)	100
Average distance (cm)—nerve to inferior pole (95% confidence interval)	2.82 (0.24)
Range	0-5
Average distance (cm)—distal arthrotomy to inferior pole	5.05 (0.67)
Range	4-7

**Figure 4:**
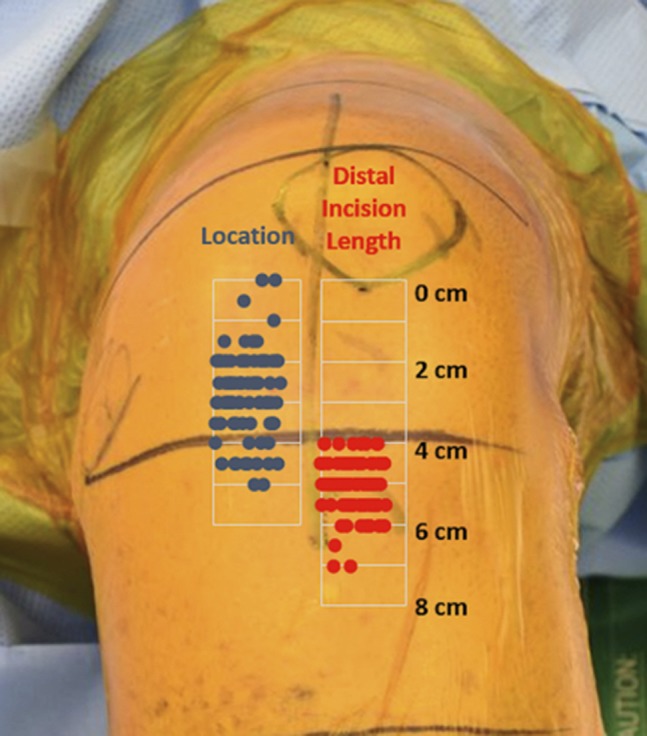
Photograph showing the location of the nerve and distal arthrotomy to the inferior pole of the patella.

**Figure 5:**
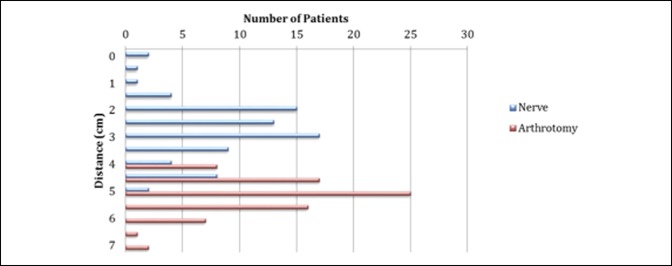
Scatter plot of the distance from the inferior pole of the patella to the infrapatellar nerve branch and end of distal arthrotomy.

The mean (SD) distance of the infrapatellar nerve to the inferior pole of the patella was 2.82 (0.24) cm, 2.9 (0.0 to 4.5) cm in men and 2.77 (0 to 5) cm in women (*P* = 0.59). The mean distance of the distal extent of the arthrotomy was 5.05 (4 to 7) cm, 5.08 cm in men and 5.03 cm in women (*P* = 0.71) (Figures 4 and 5). No notable relationships existed between location of the nerve and height (r = 0.13) or BMI (r = 0.104). No notable predictors were identified among demographics and surgical characteristics, including age, sex, height, BMI, and laterality. In all cases, the main IPNB was transected as part of the standard medial parapatellar approach.

Three patients underwent staged bilateral TKA during the study period. Comparing their left and right knees, the first patient had a nerve-to-inferior pole of the patella distance of 3 cm in both knees and an arthrotomy-to-inferior pole distance of 5.0 cm for the left knee and 4.5 cm for the right knee. The second patient had a nerve-to-inferior pole of the patella distance of 4.5 cm and 2.5 cm and an arthrotomy-to-inferior pole distance of 5.5 cm and 5.0 cm in the left and right knee, respectively. The final patient had a nerve-to-inferior pole of the patella distance of 2.0 cm and 3.5 cm and an arthrotomy-to-inferior pole distance of 4.5 cm and 5.0 cm in the left and right knee, respectively.

## Discussion

TKA has been used to reduce arthritic pain with success in most patients; however, nearly 20% are dissatisfied with their result.^3^ A cause of this dissatisfaction may be because of extra-articular neuroma formation of the IPNB.^5,17,20-22^ Previous studies evaluated painful neuroma formation and addressed this problem, with overall success.^20,23^

A study by Nahabedian and Johnson^22^ reviewed 25 patients (11 after TKA) and determined that the source of their patients' postoperative pain was neuroma formation. The diagnosis of a neuroma was made based on characteristics of the pain and lack of alternative etiology. These patients underwent selective denervation, with good to excellent results reported in 84%.^22^ Shi et al^23^ showed similar improvement in postoperative pain with selective surgical denervation after TKA with neuroma excision. This intervention has been identified as an acceptable form of treatment once a neuroma is formed.^23^ Clendenen et al^20^ identified 16 patients with persistent medial knee pain after TKA and performed ultrasound-guided nerve hydrodissection and corticosteroid injection of the IPNB. Over half of these patients reported complete or near-complete resolution of pain. In this study, the nerve was identified using ultrasonography and confirmed by eliciting paresthesias with electrical stimulation.^20^

Several studies have investigated whether the IPNB was the nerve at risk for iatrogenic injury during the approach for TKA by assessing postoperative hypesthesias around the incision. Maniar et al^24^ compared 20 patients who underwent bilateral TKAs with a midline incision on one knee and an anterolateral incision on the other knee, reporting an increased area of hypesthesias in the knee that underwent the midline incision. When comparing an anteromedial incision with an anterolateral incision, a markedly larger area of hypesthesias developed in the anteromedial incision group.^25^ Both studies demonstrated that a more medial approach denervates a larger portion of the anterior knee.

The course of the IPNB has been studied with cadaveric dissection and has been found to cross the anterior knee at a level that would place it at risk during TKA. Kartus et al^12^ reported that the IPNB crossed the anterior knee between the inferior pole of the patella and the tibial tubercle in 59 of 60 specimens, approximately 3 cm distal to the inferior pole of the patella on average. Walshaw et al^9^ noted that the IPNB crossed the patellar tendon in all 25 specimens. Kerver et al^8^ evaluated 20 embalmed cadavers and dissected the IPNB to study the zones at risk. Although low-risk zones existed, no risk-free areas around the anteromedial knee were observed.^8^

Based on cadaveric studies, the IPNB is at risk for injury during surgeries around the knee because of its anatomic location.^8-10,19,20^ However, none of these studies were prospective clinical studies describing the specific anatomic location of the nerve during primary TKA. More importantly, we were able to demonstrate that this nerve can be effectively identified by the general orthopaedic arthroplasty surgeon intraoperatively through a standard incision without assistance from anatomists or using magnification, embalming, or dye. In our series, all IPNBs were encountered and transected in every patient undergoing TKA.

This study was able to show that the IPNB was, on average, between 2.58 and 3.06 cm below the inferior pole of the patella. No demographic predictors of the location of the nerve were identified, including sex, height, and BMI.

Limitations of this study include a smaller sample size, which can affect the generalizability of the results. In addition, only the single largest branch of the infrapatellar saphenous nerve was used for measurement, whereas smaller branching nerves were not included. Significance of the smaller branches is still unknown. The nerve was also identified using clinical judgment and direct visual identification of neural fascicles. No pathologic specimens were sent for histologic confirmation.

## Conclusion

This prospective study describes the vulnerability of the IPNB during TKA because it was encountered in all incisions. This branch can be identified by the surgeon by direct visualization intraoperatively without the aid of magnification or dye. No notable difference existed in the location regarding sex, height, or BMI. The results from this study may improve preoperative patient education, increase surgeons' intraoperative awareness of the IPNB, and inspire future studies to evaluate potential techniques at the time of the primary TKA to minimize neuromatous postoperative morbidity.
